# Isolation and Expression of *NAC* Genes during Persimmon Fruit Postharvest Astringency Removal

**DOI:** 10.3390/ijms16011894

**Published:** 2015-01-15

**Authors:** Ting Min, Miao-Miao Wang, Hongxun Wang, Xiaofen Liu, Fang Fang, Donald Grierson, Xue-Ren Yin, Kun-Song Chen

**Affiliations:** 1 Laboratory of Fruit Quality Biology/The State Agriculture Ministry Laboratory of Horticultural Plant Growth, Development and Quality Improvement, Zhejiang University, Zijingang Campus, Hangzhou 310058, China; E-Mails: 11016045@zju.edu.cn (T.M.); wmm@zju.edu.cn (M.-M.W.); 0013915@zju.edu.cn (X.L.); 21216047@zju.edu.cn (F.F.); donald.grierson@nottingham.ac.uk (D.G.); akun@zju.edu.cn (K.-S.C.); 2 College of Food Science and Engineering, Wuhan Polytechnic University, Changqing Campus, Wuhan 430023, China; E-Mail: hongxunwang1977@gmail.com; 3 Plant & Crop Sciences Division, School of Biosciences, University of Nottingham, Sutton Bonington Campus, Loughborough LE12 5RD, UK

**Keywords:** persimmon, deastringency, *NAC*, low oxygen stress, transcription factor

## Abstract

*NAC* genes have been characterized in numerous plants, where they are involved in responses to biotic and abiotic stress, including low oxygen stress. High concentration of CO_2_ is one of the most effective treatments to remove astringency of persimmon fruit owing to the action of the accumulated anoxia metabolite acetaldehyde. In model plants, *NAC* genes have been identified as being responsive to low oxygen. However, the possible relationship between NAC transcription factors and persimmon astringency removal remains unexplored. In the present research, treatment with a high concentration of CO_2_ (95%) effectively removed astringency of “Mopan” persimmon fruit by causing decreases in soluble tannin. Acetaldehyde content increased in response to CO_2_ treatment concomitantly with astringency removal. Using RNA-seq and Rapid amplification of cDNA ends (RACE), six *DkNAC* genes were isolated and studied. Transcriptional analysis indicated *DkNAC* genes responded differentially to CO_2_ treatment; *DkNAC1*, *DkNAC3*, *DkNAC5* and *DkNAC6* were transiently up-regulated, *DkNAC2* was abundantly expressed 3 days after treatment, while the *DkNAC4* was suppressed during astringency removal. It is proposed that *DkNAC1/3/5/6* could be important candidates as regulators of persimmon astringency removal and the roles of other member are also discussed.

## 1. Introduction

Proanthocyanidins (PAs) are secondary metabolites and phenolic oligomers that result from the condensation of flavan-3-ol units. PAs have a variety of uses and are beneficial for human health owing to their antioxidant properties, which include anti-allergic, anti-tumor, anti-aging properties, and the prevention of cardiovascular disease [1,2,3]. Moreover, they can be used to detoxicate snake venom and have been used to adsorb radioactive compounds from nuclear waste and recycle the heavy metals from electronic waste [4,5,6,7]. Persimmon (*Diospyros kaki*) is a unique fruit that accumulates PAs in abundance [8]. Persimmon fruit can be divided into astringent and nonastringent types, and most of the commercial cultivars in China are of the astringent type [9]. Astringent persimmons accumulate abundant PAs in the fruit flesh even at maturity and soluble PAs cause astringency, which severely affects the industry and consumer acceptance [9,10]. Thus, astringency removal is critical for the persimmon industry.

Due to the importance of astringency removal for commercial production, a range of technologies has been developed, such as CO_2_ and N_2_ [11,12,13]; C_2_H_4_ and alternate freezing and thawing [14,15]. Among these, high concentration CO_2_ treatment is very commonly used and its underlying physiological and molecular mechanisms of astringency removal are much more clearly understood than for the other treatments. It has been suggested that acetaldehyde, which accumulates during anaerobic respiration induced by high CO_2_, causes soluble tannins to become insoluble during the treatment, thus reducing astringency [16]. Alcohol dehydrogenase (ADH) and Pyruvate decarboxylase (PDC) enzyme activities increased during deastringency and this was associated with acetaldehyde biosynthesis [17]. Our previous results indicated that ADH and PDC enzyme were both induced by CO_2_ and C_2_H_4_ treatments, and *DkADH1*, *DkPDC1*, *DkPDC2* and *DkPDC3* expression was up-regulated in response to both ethylene and high CO_2_ treatments [18]. When *DkPDC2* was transiently over-expressed in persimmon leaves, they showed a lower level of soluble tannins, compared with leaves infiltrated with an empty vector, suggesting that *DkPDC2* is a key gene regulating soluble tannins and astringency removal [18]. These results suggested CO_2_ driven astringency removal requires acetaldehyde metabolism, brought about by modulation of ADH and PDC enzymes and genes. However, understanding of the molecular basis of persimmon astringency removal is still limited.

In model plants, a few transcription factors have been reported to be involved in the hypoxia response, such as NAC and ethylene response factors (*ERF*) [19]. As mentioned above, persimmon fruit astringency removal by CO_2_ treatment is considered to operate mainly via the hypoxia fermentation pathway. In persimmon fruit, the only transcription factors analyzed during persimmon fruit astringency removal have been the *ERF* gene family. Recently, four *ERF* genes, *DkERF9*, *DkERF10*, *DkERF19* and *DkERF22*, were shown to manifest trans-activation of *DkADH* and *DkPDC* genes [20]. However, the relations between other transcription factors and astringency removal have rarely been reported. In addition to *ERFs*, *NAC* genes are the main transcription factors reported to be involved in the plant hypoxia response, which indicated their potential to be involved in persimmon astringency removal. The NAC designation is based on highly conserved consensus sequences in the *N*-terminal region of a Petunia gene (NAM), *Arabidopsis* ATAF1/2 and CUC2 proteins [21]. More than 100 *NAC* genes have been identified and characterized in *Arabidopsis* [22]. Among them, *ANAC019*, *ANAC055* and *ANAC072* enhanced tolerance to drought stress in *Arabidopsis* [23], and *ANAC2* is involved in response to plant hormones [24]. In addition, *ANAC102* was shown to be induced by low oxygen (0.1%), and overexpression of *ANAC102* up-regulated *ADH* expression [19]. Since treatment with a high concentration of CO_2_ triggered expression of *ADH*/*PDC* genes and acetaldehyde metabolism in persimmon, this suggested that the *Arabidopsis* low oxygen responsive transcription factors such as *ANAC102* had the potential to be involved in astringency removal, but experimental evidence for this was lacking in persimmon.

In the present research, six *DkNAC* genes were isolated from “Mopan” persimmon fruit based on differentially expressed genes (DEG) in an RNA-seq database using Rapid amplification of cDNA ends (RACE) technology. High CO_2_ (95%) treatment was applied to the fruit and *DkNAC* transcripts were analyzed during the deastringency process. Some *DkNAC* genes were found to be positively correlated with persimmon fruit deastringency, and the possible roles of these and other *DkNAC* genes are discussed.

## 2. Results and Discussion

### 2.1. Fruit Deastringency

**Figure 1:**
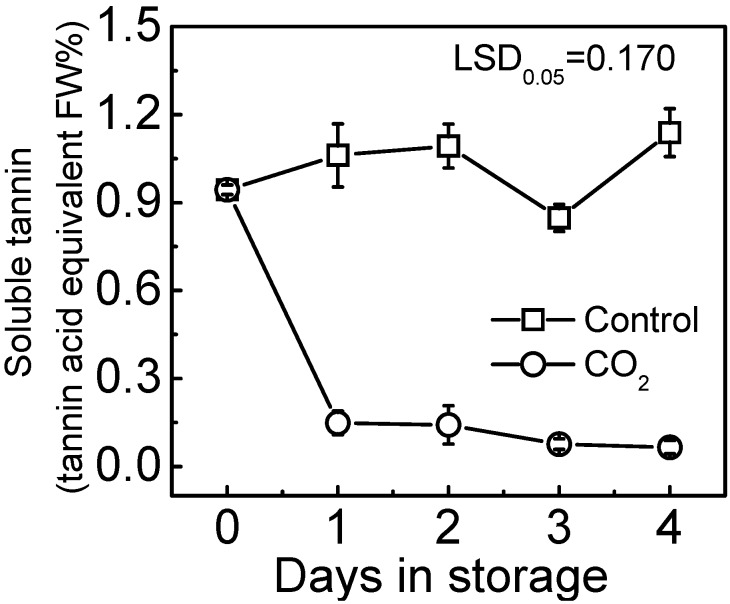
Effect of CO_2_ treatment on soluble tannin of “Mopan” fruit at 20 °C. Mature fruit were treated with CO_2_ (~95%, *v*/*v*, open circles, one day) and air (control, open squares), separately. Error bars represent standard error from three biological replicates.

**Figure 2:**
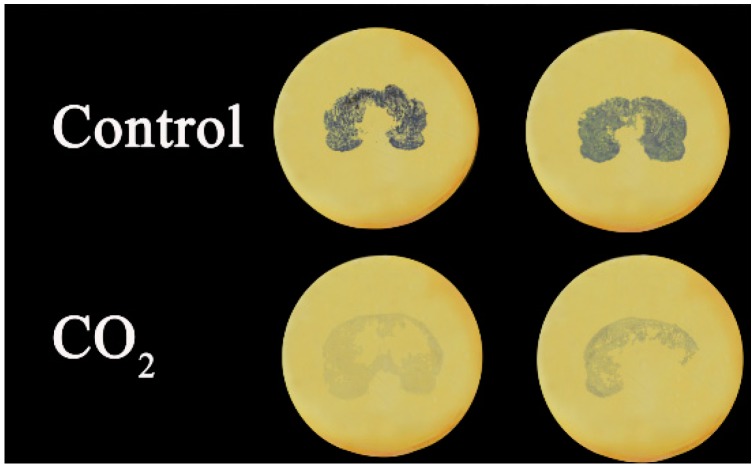
Comparison of tannin printing of control and CO_2_ treated “Mopan” fruit at 2 days in storage.

The soluble tannin content remained almost constant during storage in the control fruit, whereas in contrast, the CO_2_ treatment caused a rapid decrease in the concentration of soluble tannin from 0.917% at day 0% to 0.229% at day 1 (Figure 1). Soluble tannins content was measured both by the Folin phenol method, and also visualized by tannin printing, using filter paper soaked with 5% FeCl_2_. The extent, and location, of soluble tannin content was revealed by the tissue printing, and was substantially lower in CO_2_ treated fruit, compared with control fruit (Figure 2).

**Figure 3:**
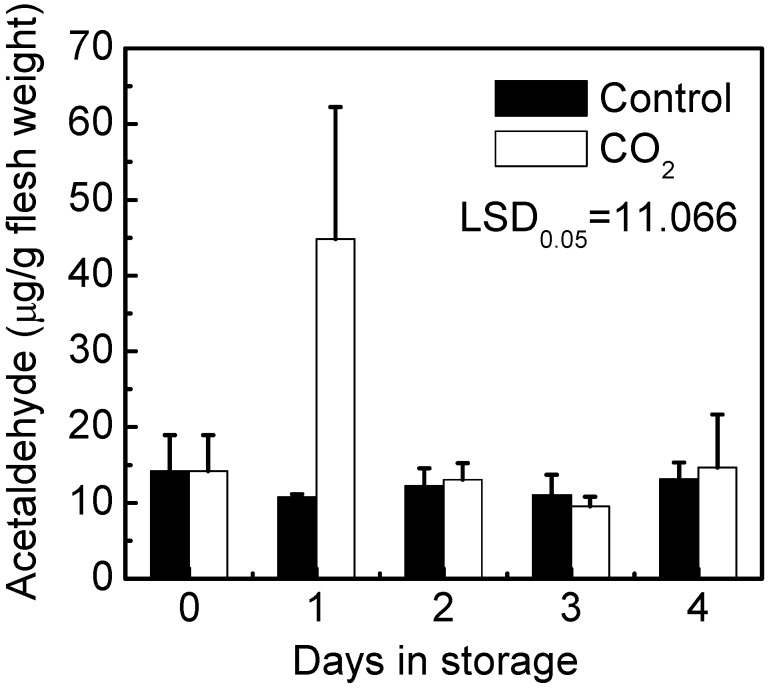
Effect of CO_2_ treatment on acetaldehyde content of “Mopan” fruit. Mature fruit were treated with CO_2_ (~95%, *v*/*v*, white bars, one day) and air (control, black bars), separately at 20 °C. Error bars represent standard error from three biological replicates.

Acetaldehyde, the main compound responsible for insolublization of soluble tannin, was also analyzed. The results indicated that there was a very obvious increase (approx. five fold) in acetaldehyde accumulation in CO_2_ treated fruit one day after the start of treatment. However, such differences were abolished, following cessation of the treatment (Figure 3).

By utilizing soluble tannin and tanning printing assays, 95% CO_2_ treatment was shown to be effective in inducing deastringency in postharvest “Mopan” cultivar persimmon. These results confirm and extend our previous findings [18] and other reports using various persimmon cultivars [11,12,18]. A burst of acetaldehyde production during deastringency treatment has been widely reported in various persimmon cultivars, such as “*Rojo Brillante*”, “*Kaki Tipo*”, “*Lycopersicon*” [25,26]. Thus, the transient accumulation of acetaldehyde further supports the role and effectiveness of 95% CO_2_ treatment on persimmon fruit deastringency.

**Figure 4:**
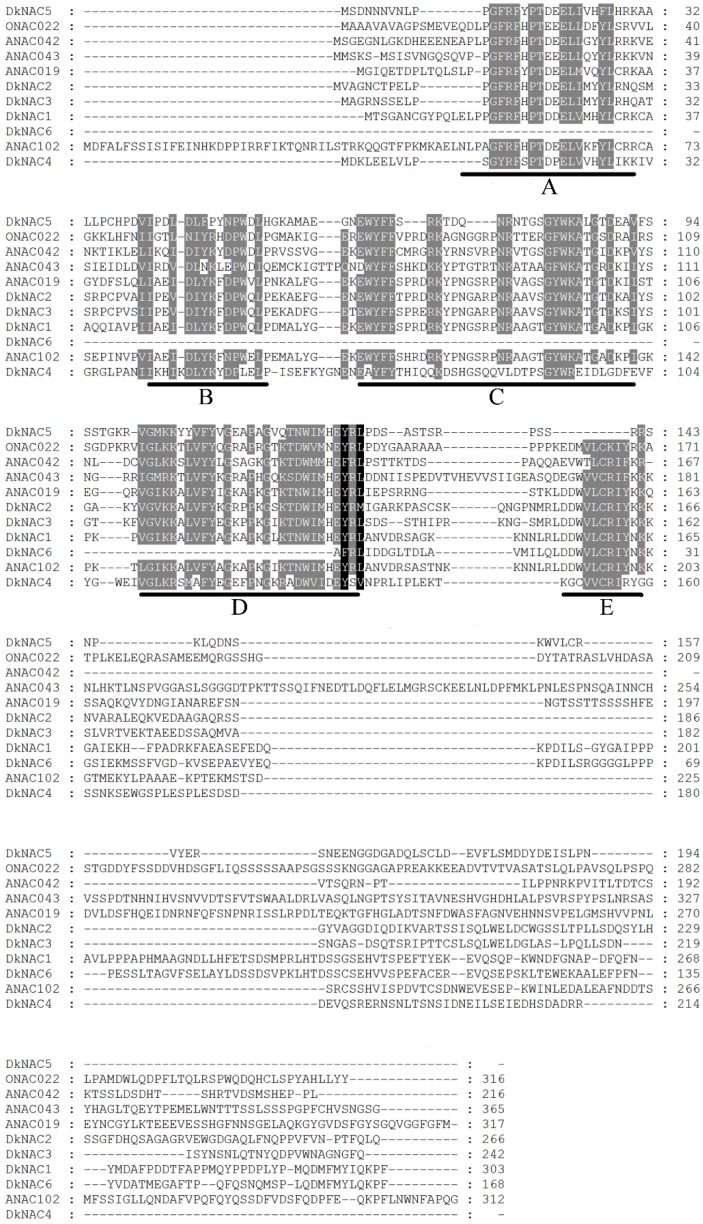
Amino acid sequence alignment of the DkNAC proteins with *Arabidopsis* and rice NAC proteins. DkNAC proteins were aligned with *Arabidopsis* ANAC019 (At1g52890.1), ANAC042 (At2g43000.1), ANAC043 (At2g46770.1), ANAC102 (AT5G63790.1) and rice ONAC022 (AK107090). Identical and similar amino acids are indicated by black and grey shading, respectively. Gaps were introduced to optimize alignment. The five highly conserved amino acid motifs (**A**–**E**) are indicated by black lines.

### 2.2. NAC Genes Isolation and Sequence Analysis

Six novel putative *NAC* genes were isolated from persimmon fruit, five of which were full-length sequences and one partial CDS, and designated as *DkNAC1-6* (GenBank accession nos. KP222303–KP222308). *DkNAC1-5* were predicted to encode proteins of 303, 266, 242, 214 and 194 amino acid (aa), respectively, however, the protein encoded by *DkNAC6* could not be predicted from the partial CDS.

Alignment analysis with the deduced proteins encoded by the *DkNAC* genes showed that they contained the NAC conserved domain in their *N*-terminal regions, which was divided into five subdomains (A–E) [27] (Figure 4). However, the *C*-terminal regions showed less similarity between different members of the NAC family (Figure 4).

**Figure 5:**
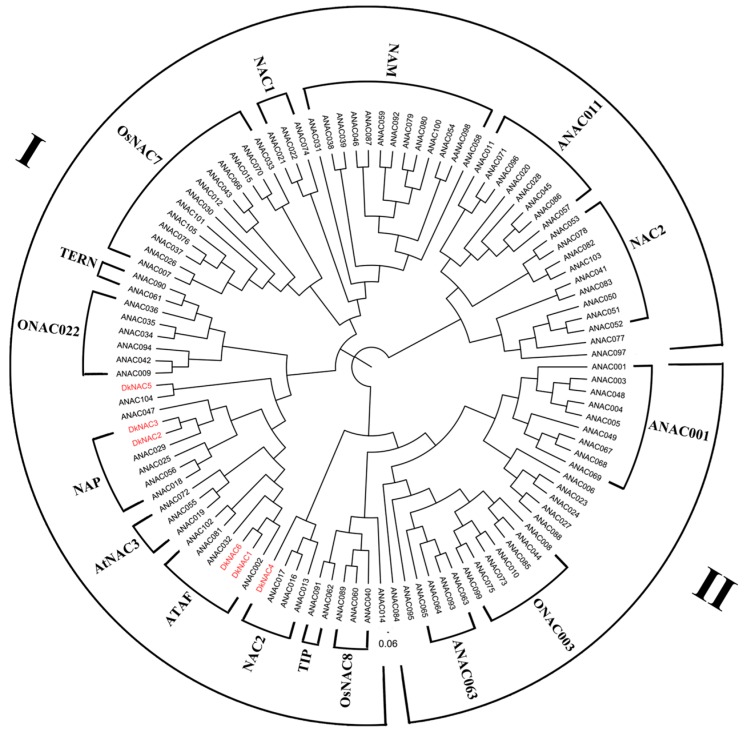
Phylogenetic tree of *NAC* genes. Persimmon *DkNAC* genes are highlighted in red. The amino acid sequences of the *Arabidopsis ERF* family were obtained from TAIR. The phylogenetic tree was constructed with figtree (version 3.1).

To examine the phylogenetic relationship between the NAC proteins in persimmon and *Arabidopsis*, a phylogenetic tree was constructed based on their translated amino acid sequences. As shown in Figure 5, *DkNAC1* and *DkNAC6* were homologous with, and closely related to *ANAC102*, which is involved in the viability of *Arabidopsis* seeds following low-oxygen treatment [19]; *DkNAC2* and *DkNAC3* share the same branch, and are closely related to *ANAC029*, which was reported to play an important role in leaf senescence in *Arabidopsis* [28]; *DkNAC4* was clustered with *ANAC013*, which has been reported to be involved in mitochondrial retrograde regulation of the oxidative stress response in *Arabidopsis* [29]; *DkNAC5* was similar to *ANAC104*, which was identified as part of the leaf senescence transcriptome [30]. Collectively, these data suggested that *DkNAC1-6* may exhibit diverse functions.

In order to investigate the possible roles of NAC transcription factors in persimmon fruit deastringency, six differentially expressed *DkNAC* genes were identified by RNA-seq data [20]. Alignment of the six DkNAC proteins showed that they shared a highly conserved *N*-terminal region, containing five consensus subdomains, which are termed the NAC domains [27]. *NAC* genes have been classified previously into two groups based on similarities of NAC domain structures in *Arabidopsis thaliana* and rice [27]. Group I proteins are composed of 14 subgroups, including TERN, ONAC022, SENU5, NAP, AtNAC3, ATAF, OsNAC3, NAC2, ANAC011, TIP, OsNAC8, OsNAC7, NAC1 and NAM, and group II can be divided into four subgroups, namely ANAC011, ONAC003, ONAC001 and ANAC063 [27]. *DkNAC1* and *DkNAC6* belong to group I (ATAF subfamily)*. ANAC102* belongs to the ATAF subgroup [19]. DkNAC2-4 also belongs to group I, whereas *DkNAC2 and DkNAC3* belong to the *NAP* subfamily and *DkNAC4* belongs to the *NAC2* subfamily and *DkNAC5* belongs to group II. Thus, phylogenetic and structural analyses indicated that *DkNAC1* and *DkNAC6* were mostly likely to be involved in persimmon deastringency, as indicated by the high similarity with *ANAC102*.

### 2.3. NAC Genes Expression

**Figure 6:**
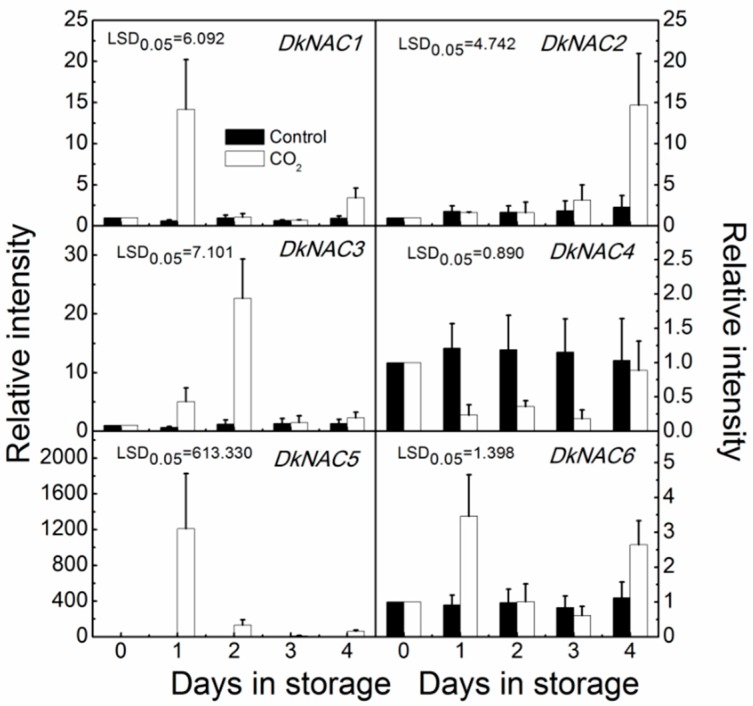
Transcriptional analysis of *DkNAC* genes. Transcripts of *DkNAC* genes were measured by real-time PCR. Fruit were treated with 95% CO_2_ for one day at 20 °C in sealed container, while control fruit was sealed in a similar container without any treatment. Day 0 fruit values were set as 1. Error bars indicate standard error from three biological replicates.

The six *NAC* genes had different expression patterns. Transcripts of *DkNAC1*, *DkNAC5* and *DkNAC6* were transiently induced by the CO_2_ treatment, peaking after one day, and the expression of *DkNAC3* peaked after two days. *DkNAC5* was the most strongly up-regulated, with its mRNA increasing in abundance approximately 1200-fold following the treatment. Unlike *DkNAC1/3/5/6*, transcripts of *DkNAC2* increased at four days (three days after treatment was stopped). Unlike the other *DkNAC* genes, the expression of *DkNAC4* was decreased during CO_2_ treatment and then recovered by day 4 (Figure 6).

One of most interesting results was that *DkNAC1*, *DkNAC3*, *DkNAC5* and *DkNAC6* were up-regulated by CO_2_ treatment, and that this occurred concomitantly with the decrease in soluble tannin. This indicates that in persimmons, expression of *DkNAC1*, *DkNAC3*, *DkNAC5* and *DkNAC6* is highly correlated with fruit deastringency. The expression pattern of *DkNAC1* and *DkNAC6* further supported the prediction from phylogenetic and structural analyses, that *ANAC102* homologs might be important regulators of the hypoxia response, which benefit deastringency in persimmon fruit. Besides *DkNAC1* and *DkNAC6*, *DkNAC3* and *DkNAC5* were also observed to increase in expression in response to 95% CO_2_ treatment; however, *DkNAC3* and *DkNAC5* belong to the NAP subfamily and group II, respectively. These results may indicate that more subfamilies might be involved in the deastringency response in persimmon fruit and more generally in the hypoxia response in plants. Although hypoxia-responsive *NAC* genes have rarely been reported, the results from the studies on *ANAC102* also indicated that additional *NAC* genes might exist for the hypoxia response, as *ANAC102* knockout lines did not show altered *ADH* gene transcription in *Arabidopsis* [19]. Taken together, results from persimmon and previous finding in *Arabidopsis* indicate that additional *NAC* genes are potentially involved in the hypoxia response in plants.

In addition to the four above-mentioned *DkNAC* genes, *DkNAC2* and *DkNAC4* expression also exhibited interesting features. Unlike the positively regulated NAC genes, *DkNAC4* mRNA levels were negatively correlated with astringency removal. Nevertheless, no repressive domain, such as an EAR motif, was observed within the coding region of DkNAC4. The increase in *DkNAC2* mRNA occurred on the third day after treatment was removed, after the fruit had lost their astringency and started to senescence. These results indicated that *DkNAC2* might be involved in fruit ripening or senescence-related processes, but not astringency removal. Similar results were also reported in other fruit, such as banana, where transcripts of *MaNAC1* and *MaNAC2* were up-regulated in banana post-harvest ripening, and it was suggested that *MaNAC* genes may be involved in banana fruit ripening via interaction with ethylene signalling components [31]. It was also reported in *Arabidopsis* siliques that *AtNAP*, a *NAC* gene whose expression increased with the progression of silique senescence, played a key role in its senescence [32].

## 3. Experimental Section

### 3.1. Materials and Treatments

The “Mopan” (astringent cultivar) persimmon fruit were harvested from a commercial orchard (Fangshan, Beijing) in 2013, with mean firmness of 47 N, and then transported to the laboratory the next day. Fruits with no defects and uniform size were chosen for treatment. CO_2_ (95%) and control treatments were performed according to our previous report [18] (Min *et al.*, 2012). After firmness measurement at each sampling point, fruit flesh was removed from the peel and shredded, then frozen with liquid nitrogen and kept in −80 °C refrigerator until required.

### 3.2. Soluble Tannins Assay and Tannin Printing

The content of soluble tannins, the most important index for astringency, was measured using Folin-Ciocalteu reagent according to the method described in our previous report [14] (Yin *et al.*, 2012).

The printing method, a convenient way of identifying persimmon astringency location and loss, was also used for the examination of soluble tannins in the fruit flesh according to [33] Hu *et al.*, (2013). Filter papers were soaked with 5% FeCl_2_ for about 5–10 min, then moved to a 60 °C oven until dry. The whole fruit was cut into two parts and printed on the processed filter paper. The black color indicated soluble tannins and the intensity of black reflected the soluble tannins content.

### 3.3. Acetaldehyde Determination

Acetaldehyde production was determined with a gas chromatograph Instrument (Agilent 6890N, Folsom, CA, USA) with a FID column (HP-INNOWAX, 0.25 mm, 30 m, 0.25 μm, Agilent J&W, Folsom, CA, USA) according to the method described previously [20] (Min *et al.*, 2014) with some modification. The injector, detector and oven temperatures were 150, 160, and 100 °C, respectively. Sec-butyl alcohol was added to each vial as an internal control. The results were calculated using standard curves for acetaldehyde and ethanol, respectively.

### 3.4. RNA Extraction and cDNA Synthesis

Total RNA was prepared according to the method used previously [18] (Min *et al.*, 2012). Traces of contaminating genomic DNA in total RNA were removed with TURBO DNase (Life Technologies, Gaithersburg, MD, USA). One microgram DNA-free RNA was used for cDNA synthesis by iScript cDNA Synthesis kit (Bio-Rad, Hercules, CA, USA) following the manufacturer’s protocol. For each sampling point, three biological replicates were used for RNA extraction.

### 3.5. Gene Isolation and Sequence Analysis

The *NAC* genes were isolated based on RNA-Seq data. RNA-Seq was performed by the Beijing Genome Institute (BGI) (Shenzhen, China) according to our previous paper [20] (Min *et al.*, 2014). Rapid amplification of cDNA ends (RACE) was chosen to amplify the 3' and/or 5' end Untranslated region (UTR) of *NAC*, using a SMART™ RACE cDNA Amplification Kit (Clontech, Palo Alto, CA, USA). The sequences of primers used for RACE are listed in Table 1. The gene sequences were translated with online software (http://web.expasy.org/translate/) and were confirmed with the BLAST methods in Genbank. A phylogenetic tree of *NAC* genes was generated using ClustalX (v 1.81) [34] and calculated using Figtree (v1.3.1, University of Edinburgh, Edinburgh, UK). The deduced amino acid sequences of homologous genes of *Arabidopsis* were obtained from TAIR (The *Arabidopsis* Information Resource).

**Table 1:** Sequences of the primers used for gene isolation.

Method Used	Gene	Primary PCR (5' to 3')	Secondary PCR (5' to 3')
3'RACE	*DkNAC1*	CAAGTTTGATCCGTGGCAACTTCCAG	TGGCTCTGTATGGAGAGAAAGAATGG
	*DkNAC2*	CCCAAGAGACCGGAAGTACCCGAAC	GGCGTCAAGAAAGCCCTCGTCTTCT
	*DkNAC3*	AGAAGATTCAAGCGCCCAGATGGTG	TACCAGGACCCAGTTTGGAATGCAG
	*DkNAC4*	CAAGAGCAGAAGTCACAAACAACTA	GAGCTGGAAGAGCTGGTTTTACC
	*DkNAC5*	TCACGCTATCTCGGTCTTCGCTCTC	GGAGCTCATCGTCCATTTCCTCCAC
	*DkNAC6*	AGTCCGGTGAGAGCGATATGATAG	GTGCCGAAGCTCCACACGGACTCG
5'RACE	*DkNAC3*	TCCTGCATTCCAAACTGGGTCCTGGT	TGTCTTGATACCCTTTGGGGGCTTTCC
	*DkNAC4*	CCCAATCTGCTCGTTTCCCGTTTGG	CAACCCAACGATTTCCCACCCATAG
	*DkNAC5*	CGTTCCCCTCCGCCATAGCTTTTCCA	GAGGGCAGCTTTGCGGTGGAGGAAA
Full clone	*DkNAC1*	CAGAGAGAGAGAGCTTGATCAGTG	CGCACCCGATTGAGAAAAT
	*DkNAC2*	TATAAATGGTGGCCGGAAAT	TCCGCACCTCGTACCTATCT
	*DkNAC3*	CAAGAAAATGGCAGGAAGAAA	GCTGATTCACTGAAACCCATT
	*DkNAC4*	CCGTCGGTACATGGAGAGAA	TGTCACATAGCCTGCTATAAAGAAGTA
	*DkNAC5*	ACGCAGAGTACATGGGGAAT	ACGGAGAAGCCTGCATAGC

### 3.6. Oligonucleotide Primers and Real-Time PCR

Oligonucleotide primers for real-time PCR analysis were designed with primer3 (v. 0.4.0, http://frodo.wi.mit.edu/cgi-bin/primer3/primer3_www.cgi). The specificity of primers was determined by melting curves and PCR products resequencing as described in [35] Yin *et al.* (2008). The sequences of oligonucleotide primers are listed in Table 2.

**Table 2:** Sequences of the primers used for real-time PCR.

Gene	Primary PCR (5' to 3')	Secondary PCR (5' to 3')
*DkNAC1*	GGTAGCATCATAAGCGTTAATCTG	CAAGAATGACCCTATTACTACCACT
*DkNAC2*	GGTGCGGATCGTAGAAACTA	ACAATTTTTGGGCCATAGGT
*DkNAC3*	GATGTGGCTTGTTAGGCTTGA	CCCACAACAATACACGTTTGTTTCA
*DkNAC4*	GCTGTATCTTTCTTGCATTGTTGAC	CAAGGGAGGAATGCCATGTA
*DkNAC5*	GCTAGCTATGCAGGCTTCTCC	ACAAACAGCGCAACTCATTT
*DkNAC6*	ACGCAGAGTACATGGGGAAT	ACGGAGAAGCCTGCATAGC

Real-time PCR was carried out with Ssofast EvaGreen Supermix kit (Bio-Rad) and CFX96 instrument (Bio-Rad) for gene expression studies according to our previous report [18] (Min *et al.*, 2012). The relative abundance of each gene was calibrated with samples from day 0 fruit. Actin, a housekeeping gene, was used as the internal control [9] (Akagi *et al.*, 2009).

### 3.7. Statistical Analysis

Origin 8.0 (OriginLab, Northampton, MA, USA) was used to prepare the figures. Statistical significance of differences was calculated with least significant difference (LSD) using DPS software (v. 3.11, Zhejiang University, Hangzhou, China).

## 4. Conclusions

In conclusion, soluble tannins and acetaldehyde assay results showed that a high concentration of CO_2_ treatment was effective in inducing deastringency of “Mopan” persimmon fruit. Six *DkNAC* genes were isolated and the transcriptional level of *DkNAC1*, *DkNAC3*, *DkNAC5* and *DkNAC6* were highly correlated with fruit deastringency. Finally, *DkNAC1/3/5/6* are identified as prime candidates for further transcriptional regulatory analysis in persimmon fruit to understand the molecular control of astringency removal.

## References

[1] Ahmad N., Gupta S., Mukhtar H. (2000). Green tea polyphenol epigallocatechin-3-gallate differentially modulates nuclear factor κB in cancer cells *versus* normal cells. Arch. Biochem. Biophys..

[2] Pataki T., Bak I., Kovacs P., Bagchi D., Das K., Tosaki A. (2002). Grape seed proanthocyanidins improved cardiac recovery during reperfusion after ischemia in isolated rat hearts. Am. J. Clin. Nutr..

[3] Serafini M., Bugianesi R., Maiani G., Valtuena S., de Santis S., Crozier A. (2003). Plasma antioxidants from chocolate. Nature.

[4] Gu H.F., Liang J., Li C.M., Zhang J., Dou H.L. (2008). Study on inhibitory effects of persimmon tannins on activities of major enzymes from several snake venoms and its mechanism. Sci. Agric. Sin..

[5] Inoue K., Kawakita H., Ohio K., Oshima T. (2006). Adsorptive removal of uranium and thorium with a crosslinked persimmon peel gel. J. Radioanal. Nucl. Chem..

[6] Parajuli D., Kawakita H., Inoue K., Ohto K., Kajiyama K. (2007). Persimmon peel gel for the selective recovery of gold. Hydrometallurgy.

[7] Xiong Y., Adhikari C.R., Kawakita H., Ohto K., Inoue K., Harada H. (2009). Selective recovery of precious metals by persimmon waste chemically modified with dimethylamine. Bioresour. Technol..

[8] Luo C., Zhang Q.L., Luo Z.R. (2014). Genome-wide transcriptome analysis of Chinese pollination-constant nonastringent persimmon fruit treated with ethanol. BMC Genomics.

[9] Akagi. T., Ikegami A., Tsujimoto T., Kobayashi S., Sato A., Kono A., Yonemori K. (2009). DkMyb4 is a Myb transcription factor involved in proanthocyanidin biosynthesis in persimmon fruit. Plant Physiol..

[10] Yonemori K., Suzuki Y. (2008). Differences in three-dimensional distribution of tannin cells in flesh tissue between astringent and non-astringent type persimmon. Acta Hortic..

[11] Arnal L., Del-Río M.A. (2003). Removing astringency by carbon dioxide and nitrogen-enriched atmospheres in persimmon fruit cv. “*Rojo brillante*”. J. Food Sci..

[12] Salvador A., Arnal L., Besada C., Larrea V., Quiles A., Pérez-Munuera I. (2007). Physiological and structural changes during ripening and deastringency treatment of persimmon fruit cv. “*Rojo Brillante*”. Postharvest Biol. Technol..

[13] Yamada M., Taira S., Ohtsuki M., Sato A., Iwanami H., Yakushiji H., Wang R.Z., Yang Y., Li G.C. (2002). Varietal differences in the ease of astringency removal by carbon dioxide gas and ethanol vapor treatments among oriental astringent persimmons of Japanese and Chinese origin. Sci. Hortic..

[14] Yin X.R., Shi Y.N., Min T., Luo Z.R., Yao Y.C., Xu Q., Ferguson I.B., Chen K.S. (2012). Expression of ethylene response genes during persimmon fruit astringency removal. Planta.

[15] Taira S., Ono M., Otsuki M. (1998). Effects of freezing rate on astringency reduction in persimmon during and after thawing. Postharvest Biol. Technol..

[16] Matsuo T., Ito S. (1997). On mechanisms of removing astringency in persimmon fruits by carbon dioxide treatment I. Some properties of the two processes in the de-astringency. Plant Cell Physiol..

[17] Tamura F., Tanabe K., Itai A., Hasegawa M. (1999). Characteristics of acetaldehyde accumulation and removal of astringency with ethanol and carbon dioxide treatments in “*Saijo*” persimmon fruit. J. Jpn. Soc. Hortic. Sci..

[18] Min T., Yin X.R., Shi Y.N., Luo Z.R., Yao Y.C., Grierson D., Ferguson I.B., Chen K.S. (2012). Ethylene-responsive transcription factors interact with promoters of *ADH* and *PDC* involved in persimmon (*Diospyros kaki*) fruit de-astringency. J. Exp. Bot..

[19] Christianson J.A., Wilson I.W., Llewellyn D.J., Dennis E.S. (2009). The low-oxygen-induced NAC domain transcription factor *ANAC102* affects viability of *Arabidopsis* seeds following low-oxygen treatment. Plant Physiol..

[20] Min T., Fang F., Ge H., Shi Y.N., Luo Z.R., Yao Y.C., Grierson D., Yin X.R., Chen K.S. (2014). Two novel anoxia-induced ethylene response factors that interact with promoters of deastringency-related genes from persimmon. PLoS One.

[21] Aida M., Ishida T., Fukaki H., Fujisawa H., Tasaka M. (1997). Genes involved in organ separation in *Arabidopsis*: An analysis of the *cup-shaped cotyledon* mutant. Plant Cell.

[22] Nuruzzaman M., Manimekalai R., Sharon A.M., Satoh K., Kondoh H., Ooka H., Kikuchi S. (2010). Genome-wide analysis of NAC transcription factor family in rice. Gene.

[23] Tran L.S.P., Nakashima K., Sakuma Y., Simpson S.D., Fujita Y., Maruyama K., Fujita M., Seki M., Shinozaki K., Yamaguchi-Shinozaki K. (2004). Isolation and functional analysis of *Arabidopsis* stress-inducible NAC transcription factors that bind to a drought-responsive *cis*-element in the *early responsive to dehydration stress 1* promoter. Plant Cell.

[24] He X.J., Mu R.L., Cao W.H., Zhang Z.G., Zhang J.S., Chen S.Y. (2005). *AtNAC2*, a transcription factor downstream of ethylene and auxin signaling pathways, is involved in salt stress response and lateral root development. Plant J..

[25] Salvador A., Cuquerella J., Martínez-Jávega J.M., Monterde A., Navarro P. (2004). 1-MCP preserves the firmness of stored persimmon “Rojo Brillante”. J. Food Sci..

[26] Hribar J., Zavrtanik M., Simčič M., Vidrih R. (2000). Changes during storing and astringency removal of persimmon fruit (*Diospyros Kaki* L.). Acta Aliment..

[27] Ooka H., Satoh K., Doi K., Nagata T., Otomo Y., Murakami K., Matsubara K., Osato N., Kawai J., Carninci P. (2003). Comprehensive analysis of NAC family genes in *Oryza sativa* and *Arabidopsis thaliana*. DNA Res..

[28] Guo Y.F., Gan S.S. (2006). AtNAP, a NAC family transcription factor, has an important role in leaf senescence. Plant J..

[29] Clercq I.D., Vermeirssen V., van Aken O., Vandepoele K., Murcha M.W., Law S.R., Inzé A., Ng S., Ivanova A., Rombaut D. (2013). The membrane-bound NAC transcription factor ANAC013 functions in mitochondrial retrograde regulation of the oxidative stress response in *Arabidopsis.*. Plant Cell.

[30] Guo Y., Cai Z., Gan S. (2004). Transcriptome of *Arabidopsis* leaf senescence. Plant Cell Environ..

[31] Shan W., Kuang J.F., Chen L., Xie H., Peng H.H., Xiao Y.Y., Li X.P., Chen W.X., He Q.G., Chen J.Y. (2012). Molecular characterization of banana NAC transcription factors and their interactions with ethylene signalling component EIL during fruit ripening. J. Exp. Bot..

[32] Kou X.H., Watkins C.B., Gan S.-S. (2012). *Arabidopsis AtNAP* regulates fruit senescence. J. Exp. Bot..

[33] Hu Q., Luo C., Zhang Q.L., Luo Z.R. (2013). Isolation and characterization of a laccase gene potentially involved in proanthocyanidin polymerization in oriental persimmon (*Diospyros kaki* Thunb.) fruit. Mol. Biol. Rep..

[34] Thompson J.D., Gibson T.J., Plewniak F., Jeanmougin F., Higgins D.G. (1997). The ClustalX windows interface: Flexible strategies for multiple sequence alignment aided by quality analysis tools. Nucleic Acids Res..

[35] Yin X.R., Chen K.S., Allan A.C., Wu R.M., Zhang B., Lallu N., Ferguson I.B. (2008). Ethylene-induced modulation of genes associated with the ethylene signalling pathway in ripening kiwifruit. J. Exp. Bot..

